# Postmastectomy locoregional recurrence and recurrence-free survival in breast cancer patients

**DOI:** 10.1186/1477-7819-8-30

**Published:** 2010-04-17

**Authors:** Ali Arab Kheradmand, Neda Ranjbarnovin, Zahra Khazaeipour

**Affiliations:** 1 Associated professor of Plastic & Reconstruction Surgery, Cancer Institute, Imam Khomeini Hospital Complex, Tehran University of Medical Sciences, Tehran, Iran; 2 Researcher, Research Development Center, Imam Khomeini Hospital Complex, Tehran University of Medical Sciences, Tehran, Iran; 3 Preventive & Community Medicine. Research Development Center, Imam Khomeini Hospital Complex, Tehran University of Medical Sciences, Tehran, Iran

## Abstract

**Background:**

One essential outcome after breast cancer treatment is recurrence of the disease. Treatment decision is based on assessment of prognostic factors of breast cancer recurrence. This study was to investigate the prognostic factors for postmastectomy locoregional recurrence (LRR) and survival in those patients.

**Methods:**

114 patients undergoing mastectomy and adjuvant radiotherapy in Cancer Institute of Tehran University of Medical Sciences were retrospectively reviewed between 1996 and 2008. All cases were followed up after initial treatment of patients with breast cancer via regular visit (annually) for discovering the LRR. Cumulative recurrence free survival (RFS) was determined using the Kaplan-Meier method, with univariate comparisons between groups through the log-rank test. The Cox proportional hazards model was used for multivariate analysis.

**Result:**

The median follow up time was 84 months (range 2-140). Twenty-three (20.2%) patients developed LRR. Cumulative RFS rate at 2.5 years and 5 years were 86% (95%CI, 81-91) and 82.5% (95%CI, 77-87) respectively. Mean RFS was 116.50 ± 4.43 months (range, 107.82 - 125.12 months, 95%CI). At univariate and multivariate analysis, factors had not any influence on the LRR.

**Conclusion:**

Despite use of adjuvant therapies during the study, we found a LRR rate after mastectomy of 20.2%. Therefore, for patients with LRR without evidence of distant disease, aggressive multimodality therapy is warranted.

## Background

Breast cancer is the main cause of death that affects women worldwide [1]. Women who have been diagnosed with breast cancer and have completed initial treatments remain at risk for recurrent cancer [2,3]. Surgery combined with radiotherapy has been the typical treatment for the breast cancer in order to control loco-regional disease [4-6]. To avoid recurrence from micrometastasis, hormone- or chemotherapy adjuvant treatments are often prescribed.

In the previous reports, the 10-year local recurrence rates after modified radical mastectomy (MRM) are around 12% to 27% [7-10]. The locoregional recurrence (LRR) rate can reach as high as 30% in some studies [11-13].

Several studies have reported that young age [14,15], large tumors [16], multiple tumors [17], positive tumor margins [18], axillary lymph node involvement [16], extranodal extension [16], extensive ductal carcinoma-in-situ [19,20] and high nuclear grade [21] are risk factors for LRR.

Incidence and outcomes data of LRR after mastectomy are limited by heterogeneous study populations and the different time period studies. Since it is important to know the prognostic factors related with LRR and recurrence-free survival (RFS) of a population of breast cancer patients, we retrospectively investigated the recurrence and survival in patients with breast cancer after MRM. We mainly analyzed the prognostic factors related with LRR to identify the subgroup of patients with higher risk of recurrence for selective treatment (the use of more effective surgical interventions and/or adjuvant chemo- or radiotherapy).

## Methods

### Patients

To evaluate the risk of post-surgery recurrence of breast cancer, a historical cohort study was designed. The cases included in this study were selected from the female patients with breast cancer who had received MRM and adjuvant radiotherapy from 1996 to 2008 in Cancer Institute of Imam Khomeini hospital complex. One of major teaching hospitals of Tehran University of Medical Sciences in Iran. Unfortunately patients' records of our center were incomplete. There were 800 complete records that one out of 7 was reviewed. A total of 114 patients were enrolled into this study via systematic random sampling. All cases were followed up (median 84; range 2-140 months) after initial treatment of patients with breast cancer via regular visit (annually) for discovering the LRR. In addition to routine clinical examination, disease assessment included mammography, chest x ray and liver ultrasonography.

All patients received postoperatively adjuvant chemotherapy using CMF regimens (cyclophosphamide, methotrexate, and fluorouracil). Hormonal therapy was given to Sixty-six (57.9%) of the 114 patients. Hormonal therapy was given to all patients with estrogen receptor (ER)-positive or progesterone receptor (PR)-positive tumors by biochemical assay or immunohistochemistry. All patients received postoperatively adjuvant radiotherapy which was usually performed intermittently between courses of chemotherapy. Postmastectomy radiotherapy to the chest wall was only given to high-risk groups showing locally advanced primary tumor and/or metastatic axillary lymph nodes. Irradiation of the axilla, parasternal, subclavicular and supraclavicular lymph node regions was also restricted to these high-risk groups.

Cobalt-60 ray was used. The target sites for radiation always included supraclavicular/apical axillary regions. The radiation dose was DT46-50 Gy in conventional fractionations. The 6th edition of the TNM staging system of the American Joint Committee on Cancer (AJCC) was used. The histologic grade of the tumors was scored according to the system of Bloom and Richardson [22].

Patients with distant metastasis detected at the time of diagnosis and those that their surgical margins were positive for carcinoma were excluded.

The variables considered in these patients were age, weight, lymph node involvement, size, stage, grade and pathology of the tumor using operative and pathology records. The slides were reviewed by one pathologist.

LRR defined as LRR not predated or followed by distant metastases within 6 weeks [23]. The RFS was counted from the beginning date of surgery. The event endpoint of RFS was the appearance of LRR of tumors in the chest wall, supraclavicular lymph nodes, axillary lymph nodes, subclavicular lymph nodes and internal mammary lymph nodes. All the LRR was confirmed by the pathological biopsies. This study was approved by the medical ethics committee of Tehran University of Medical Sciences. We had no financial support (grants and funds) for study.

### Statistical analysis

SPSS 16 software was used for statistical analysis.

Cumulative RFS was determined by using the Kaplan-Meier method, with univariate comparisons between groups through the log-rank test. The Cox proportional hazards model was used for multivariate analysis.

All P-values were tested by two-tailed test, where < 0.05 indicated statistically significant.

## Result

The total number of patients included in our study was 114. Median age at surgery was 45 years (range, 26 - 90). Twenty-three (20.2%) patients developed locoregional recurrences. The median follow up time was 84 months (range 2-140).

In the location of 23 cases with LRR, most (91.29%) were seen at chest wall, 4.34% at internal mammary lymph nodes and 4.34% at axillary lymph nodes (Table 1). The median time to recur was 44 months (range, 2 months to 30 years).

**Table 1:** Distribution of loco regional recurrence sites in breast cancer patients.

Site of recurrence	Frequency	Percentage
Chest wall	21	91.29
Internal mammary	1	4.34
lymph node		
Axillary lymph node	1	4.34
Supraclavicular lymph node	0	0
Subclavicular lymph node	0	0
Total	23	100

Patients less than 30 years had the lowest rates of LRR (4.34%) and those with 30-40 years had the highest rates (30.43%). Patients with weight of ≤ 50 kg, 90 kg ≤ had the lowest rates of LRR (4.34%), those with weight of 60-70 kg had the highest rates (34.78%). Patients with tumors ≤ 2 cm had the lowest rates of LRR (21.7%), those with tumors 2 to 5 cm had intermediate rates (34.7%), and those with tumors ≥ 5.0 cm had the highest rates (43.4%). Patients with 4-9 lymph nodes had the highest rates of LRR (52.17%) and those with 10 or more lymph node had the lowest rates (8.6%). Patients with stage I had the lowest rates of LRR (4.34%) and those with stage III had the highest rates (69.56%). Patients with grade 1 had the lowest rates of LRR (8.6%) and those with grade 2 had the highest rates (47.82%). Among the recurrent tumors, 21 (91.30%) were invasive ductal carcinoma and 2 (8.7%) were invasive lobular carcinoma.

### Univariate survival analysis

**Figure 1:**
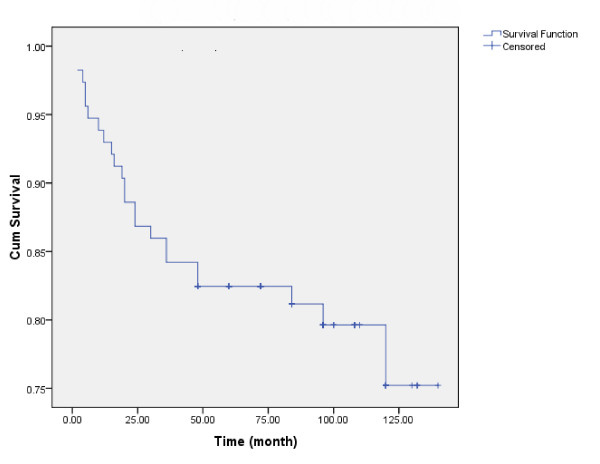
**Locoregional recurrences free survival of women with breast cancer**.

Kaplan-Meier estimates of cumulative RFS rate at 2.5 years (with 95% confidence intervals [CIs]) was 86% (81-91). At 5 year was 82.5% (77-87). Mean RFS was 116.50 ± 4.43 months (range, 107.82 - 125.12 months, 95%CI). (Fig. 1) The clinicopathological variables tested in the univariate analysis are shown in Table 2. Age, weight, tumor size, nodal status, stage, grade and histology of tumor were shown no influence on the10-year RFS rate (p > 0.05).

**Table 2:** Results of the univariate and multivariate analysis for loco regional cecurrence, according to patient and tumor characteristics.

Characteristics	Univariate			Multivariate		
	
	HR	95% CI	P value	HR	95% CI	P value
**Age (year)**	1.0	0.97-1.03	0.99	0.99	0.96-1.03	0.87
**Weight**	0.97	0.93-1.02	0.3	0.97	0.92-1.03	0.4
**Tumor size**						
*T1 = ≤ 2 cm						
T2 = 2-5 cm	0.49	0.16-1.5	0.21	0.4	0.11-1.43	0.16
T3 = > 5 cm	0.87	0.29-2.55	0.8	0.57	0.15-2.09	0.39
**Nodal Status**						
*N 0 = no involvement						
N 1-3	0.81	0.21-3.02	0.75	0.32	0.05-1.91	0.21
N 4-9	1.52	0.48-4.79	0.46	0.26	0.02-3.23	0.3
N ≥ 10	0.51	0.11-2.28	0.37	0.09	0.007-1.37	0.08
**Stage**						
*S1						
S2	0.95	0.11-7.97	0.96	3.55	0.24-51.72	0.35
S3	1.52	0.2-11.55	0.68	13.18	0.35-485.9	0.16
**Grade**						
*G1						
G2	0.98	0.21-4.43	0.98	1.004	0.20-4.96	0.99
G3	1.18	0.25-5.38	0.83	1.14	0.23-5.48	0.87
**Histologic type**						
* Ductal						
Lobular	1.21	0.28-5.2	0.78	1.5	0.29-7.53	0.62

*The base groups in Cox analysis are T1, N 0, S1, G1 and Ductal carcinoma.

Overall recurrence rates showed peaks at 5-20 month (5 and 12%, respectively).

Table 3 shows 10-year RFS rates of patients.

**Table 3:** 10-year RFS rates of loco regional recurrence according to patient and tumor characteristics

Characteristics	n	10-year RFS (%)
**Age (year)**		
30 ≥	4	75%
31-40	33	78%
41-50	25	81%
51-60	29	39%
60<	23	73%
**Weight**		
50 ≥	2	5%
51-60	11	36%
61-70	40	78%
71-80	43	78%
81-90	15	86%
90<	3	66%
**Tumor size**		
T1 = ≤ 2 cm	18	69%
T2 = 2-5 cm	55	85%
T3 = > 5 cm	41	63%
**Nodal Status**		
N 0	20	8%
N 1-3	30	82%
N 4-9	36	60%
N ≥ 10	28	89%
**Stage**		
S1	5	8%
S2	40	85%
S3	69	68%
**Grade**		
G1	10	80%
G2	58	79%
G3	46	68%
**Histology**		
Ductal	105	75%
Lobular	9	77%

*The base groups in Cox analysis are T1, N 0, S1, G1 and Ductal carcinoma.

### Multivariate survival analysis

According to the results of the multivariate Cox proportional hazards survival analysis, age, weight, tumor size, nodal status, stage, grade and histology of tumor were not significant predictors of LRR after MRM (Table 2).

## Discussion

In this study, the median follow up time was 7 years and the LRR rate was 20.2%, a rate similar to those reported for mastectomy performed in large prospective randomized trials. In those trials, local recurrence rate for patients treated with mastectomy ranged from 2% to 19%. The broad range of follow-up time in these studies (6-19 years) may account for the range of recurrence rate [24-26]. The 15-year LRR rate in 276 patients included in Danish Breast Cancer Cooperative Group (DBCG) 82b and 82c trials was found to be 27% [10].

In the present study, most recurrence of breast cancer occurred within 5-20 months. These results support previous data from Saphner et al. who identified a peak of recurrences at 2 years in a large cohort of patients (n = 3,585) enrolled in 7 Eastern Cooperative Oncology Group studies of postoperative adjuvant therapy [27].

Similar to previous studies [7,28], in the location of recurrences, chest wall was most often, taking up to 91.29%.

In this study, RFS rate at 5 - years was 82.5%. Overgaard et al. reported better 5 - year overall survival (OS) and disease-free survival (DFS) rates (72% and 61%) [29]. Ragaz et al. reported 5-year-OS and DFS rates of 60% and 47% [30].

Many reports suggested that premenopausal and younger age at breast cancer diagnosis were unfavorable prognostic factors for locoregional control and survival [8,9,31]. In the study of Mansell et al, large tumour size, high grade, involvement of more than 3 axillary nodes and the presence of lymhovascular invasion were highly significant independent predictors of recurrence within 2.5 years (P\0.001) [32]. In the BIG 1-98 trial, significant predictors of early recurrence in multivariate analysis also included tumor size and grade and node positivity [33] and in the study of Komoike et al [34], risk factors of ipsilateral breast tumor recurrence were younger age, positive margin status and omission of postoperative irradiation.

Additional factors found to be independent predictors of early recurrence include low ER positivity and human epidermal growth factor receptor 2 (HER2) overexpression/amplification [33,35].

In this report at univariate and multivariate analysis, none of factors reached statistical significance to predict LRR. Patients with tumors ≥ 5.0 cm, 4-9 involved nodes, stage III, grade 2 and ductal tumor were at increased risk of LRR. But no statistical difference was found in our group of patients.

About the influence of diet on breast cancer prognosis, the Women's Intervention Nutrition Study found that a low-fat diet reduced breast cancer recurrence [36], whereas the Women's Health Eating and Lifestyle Study reported that a diet high in vegetables, fruits, and fiber and low in total fat did not reduce recurrence or mortality [37]. A growing body of evidence suggests that patients with higher body mass index (BMI) have been found to have a higher risk of recurrence [38,39].

As reported in another studies [40,41], Durna et al [42] found that women who used hormone replacement therapy (HRT) after diagnosis of breast cancer had a significantly lower risk of cancer recurrence or new breast cancer than women who did not use HRT (RR, 0.62). In this study, we did not evaluate these factors and recommend evaluating in the future.

Number of all cases in this study was limited and we reviewed only complete records and we do not know anything about incomplete records. These were limitations of this study. We hope that more cases accumulation let better comparison in later studies. Also, our results confirmed the previous studies indicated that postoperative adjuvant radiotherapy is mainly applied for patients with four or more metastatic axillary lymph nodes and those with primary tumors at stage T3 or above, who have higher risk of locoregional recurrence.

## List of abbreviations

LRR: loco regional recurrence; MRM: modified radical mastectomy; CMF: cyclophosphamide: methotrexate: and fluorouracil; ER: estrogen receptor; PR: progesterone receptor; AJCC: American Joint Committee on Cancer; RFS: recurrence - free survival; DBCG: Danish Breast Cancer Cooperative Group; OS: overall survival; DFS: disease-free survival; HRT: hormone replacement therapy.

## Competing interests

There is no conflict of interest and any financial and personal relationships with other people or organisations in our study. This study was approved by the medical ethics committee of Tehran University of Medical Sciences.

## Authors' contributions

AA participated in the design of the study and conceived of the study. NR drafted the manuscript and acquisition of data and coordination. ZK performed the statistical analysis.
